# Genetic Inactivation of Pyruvate Dehydrogenase Kinases Improves Hepatic Insulin Resistance Induced Diabetes

**DOI:** 10.1371/journal.pone.0071997

**Published:** 2013-08-05

**Authors:** Rongya Tao, Xiwen Xiong, Robert A. Harris, Morris F. White, Xiaocheng C. Dong

**Affiliations:** 1 Department of Biochemistry and Molecular Biology, Indiana University School of Medicine, Indianapolis, Indiana, United States of America; 2 Richard Roudebush Veterans Affairs Medical Center, Indianapolis, Indiana, United States of America; 3 Division of Endocrinology, Children's Hospital Boston, Harvard Medical School, Boston, Massachusetts, United States of America; Boston University School of Medicine, United States of America

## Abstract

Pyruvate dehydrogenase kinases (PDK1-4) play a critical role in the inhibition of the mitochondrial pyruvate dehydrogenase complex especially when blood glucose levels are low and pyruvate can be conserved for gluconeogenesis. Under diabetic conditions, the *Pdk* genes, particularly *Pdk4*, are often induced, and the elevation of the *Pdk4* gene expression has been implicated in the increased gluconeogenesis in the liver and the decreased glucose utilization in the peripheral tissues. However, there is no direct evidence yet to show to what extent that the dysregulation of hepatic *Pdk* genes attributes to hyperglycemia and insulin resistance *in vivo*. To address this question, we crossed *Pdk2* or *Pdk4* null mice with a diabetic model that is deficient in hepatic insulin receptor substrates 1 and 2 (Irs1/2). Metabolic analyses reveal that deletion of the *Pdk4* gene had better improvement in hyperglycemia and glucose tolerance than knockout of the *Pdk2* gene whereas the *Pdk2* gene deletion showed better insulin tolerance as compared to the *Pdk4* gene inactivation on the *Irs1/2* knockout genetic background. To examine the specific hepatic effects of Pdks on diabetes, we also knocked down the *Pdk2* or *Pdk4* gene using specific shRNAs. The data also indicate that the *Pdk4* gene knockdown led to better glucose tolerance than the *Pdk2* gene knockdown. In conclusion, our data suggest that hepatic Pdk4 may be critically involved in the pathogenesis of diabetes.

## Introduction

Mitochondrial pyruvate dehydrogenase complex (PDC) plays an essential role in glucose metabolism by converting pyruvate to acetyl-CoA in glycolysis [1]. The activity of PDC is not only allosterically regulated by acetyl-CoA and NADH, but also by covalent modifications such as phosphorylation that is controlled by four pyruvate dehydrogenase kinases (Pdks) and two pyruvate dehydrogenase phosphatases (Pdps) [1]. Pdks have differential tissue distribution: Pdk1 is abundant in the heart and is expressed at a low level in other organs; Pdk2 is ubiquitously expressed in most tissues; Pdk3 is abundant in testis and is expressed at a low level in other organs; Pdk4 is highly expressed in the heart and skeletal muscle and is also expressed at an intermediate level in the liver, lung, and kidney [2]–[6]. Among these Pdks, Pdk4 is highly inducible by starvation and it is also elevated under insulin resistance [6]–[15]. Systemic *Pdk4* knockout leads to hypoglycemia after the prolonged starvation [16]. In contrast, *Pdk2* null mice only manifest a moderate reduction in blood glucose under non-fasted conditions [17]. When challenged by a high-fat diet, *Pdk4* knockout mice exhibit lower blood glucose levels and better glucose tolerance relative to the control wild-type mice [18].

The *Pdk4* gene expression can be suppressed by insulin under normal physiological conditions [10]. Insulin receptor substrates (Irs) play an essential role in the insulin signal transduction through a direct mediation of insulin receptor activities [19]. There are four *Irs* genes in mammals, and among them, *Irs1* and *Irs2* are ubiquitously expressed. Mouse genetic data have shown that deletion of *Irs1* and *Irs2* genes in the mouse liver (IrsLDKO) causes severe insulin resistance and early onset of diabetes [20], [21]. It is also noticed that *Pdk4* gene expression is highly induced in the liver of the IrsLDKO mice due to the impairment of insulin signaling [20]; however, it is not clear that, to what extent, the elevated Pdk4 contributes to the diabetes in the IrsLDKO mice. To address this question, we genetically inactivated *Pdk2* or *Pdk4* in the IrsLDKO mice. Our results indicate that Pdk4 indeed plays a more significant role in the development of hyperglycemia and glucose intolerance in this hepatic insulin resistance model.

## Materials and Methods

### Animals

*Irs1* and *Irs2* floxed mice and *Pdk2/4* null mice were generated as previously described [16], [17], [20]. Transgenic mice that carry a Cre coding sequence plus the Albumin gene promoter were purchased from the Jackson Laboratory. For insulin stimulation, animals were anesthetized before a bolus of 5 units of insulin (human regular insulin — humulin R, Eli Lilly) was injected via vena cava for 3 min.

### Ethics statement

All procedures were performed in accordance with the Guide for Care and Use of Laboratory Animals of the National Institutes of Health and were approved by the Institutional Animal Use and Care Committee of Indiana University School of Medicine (study 10322).

### Blood chemistry and metabolic analysis

Blood glucose levels were measured using a glucose meter under *ad libitum* (fed) or overnight 16-hour fasting. Serum insulin was measured using commercial assay kits (ALPCO). Glucose and insulin tolerance tests were performed as previously described [22], and 2 g glucose and 1 unit human regular insulin per kg body weight were used, respectively.

### Immunoblot analysis

Liver tissue was homogenized in the lysis buffer (50 mM Hepes, pH 7.5, 150 mM NaCl, 10% Glycerol, 1% Triton X-100, 1.5 mM MgCl_2_, 1 mM EGTA, 10 mM Sodium Pyrophosphate, 100 mM Sodium Fluoride and freshly added 100 uM Sodium Vanadate, 1 mM PMSF, 10 ug/ml Aprotinin, and 10 ug/ml Leupeptin). Protein extracts were resolved on an SDS-PAGE gel and transferred to nitrocellulose membrane. Proteins were probed using the following antibodies: Irs1 and Irs2 (Millipore), Pdk2, Pdk4, β-actin and Actinin (Santa Cruz Biotechnology), total and phosphorylated Akt and Erk (Cell Signaling Technology). Protein signals were detected by incubation with HRP-conjugated secondary antibodies, followed by ECL detection reagent (Thermo Fisher Scientific Inc.).

### Adenovirus-mediated gene knockdown in vivo

Gene-specific shRNAs were designed using the BLOCK-iT RNAi Designer (Invitrogen) and cloned using a BLOCK-iT system (Invitrogen). The target template sequences are the followings: shGFP, 5′-GCATCAAGGTGAACTTCAAGA-3′; shPdk2, 5′-GGCTCTTCAGCTACATGTACT-3′; and shPdk4, 5′-GGAAGGAATCAAAGCACTTTA-3′. Adenoviruses were prepared following the standard procedure. Mice were injected with adenoviruses (1×10^9^ pfu/animal) via tail vein as previously described [23]. Three days post-injection, glucose tolerance tests were performed. Five days post-injection, insulin tolerance tests were performed. On day 7 post-injection, animals were fasted overnight for 16 hours before tissues were collected for further analysis.

### Real-time PCR

Liver RNA isolation was performed as previously described (6). Quantitative RT-PCR (RT-qPCR) was performed in two steps: first, cDNA was synthesized using a cDNA synthesis kit (Applied Biosystems Inc.); second, cDNA was analyzed by real-time PCR using SYBR Green Master Mix (Promega). Primer sequences for the specific genes are as follows: Ppia forward 5′-CACCGTGTTCTTCGACATCA-3′; Ppia reverse 5′-CAGTGCTCAGAGCTCGAAAGT-3′; Pdk2 forward 5′- TGGAAAGCTCCGAGTTCAGT; Pdk2 reverse 5′- GGAGACTGGCACTCACCACT-3′; Pdk4 forward 5′- GATTGACATCCTGCCTGACC-3′; Pdk4 reverse 5′- CATGGAACTCCACCAAATCC-3′; Pck1 forward ATCATCTTTGGTGGCCGTAG; Pck1 reverse TGATGATCTTGCCCTTGTGT; G6pc forward TCGGAGACTGGTTCAACCTC; G6pc reverse TCACAGGTGACAGGGAACTG.

### Statistics

Data are presented as means ± SEM. Student's t-test (2-way) was performed to test significance between two groups. *P*<0.05 was considered as a statistical significance.

## Results

### Inactivation of *Pdk2* or *Pdk4* improves glucose homeostasis in IrsLDKO mice

**Figure 1:**
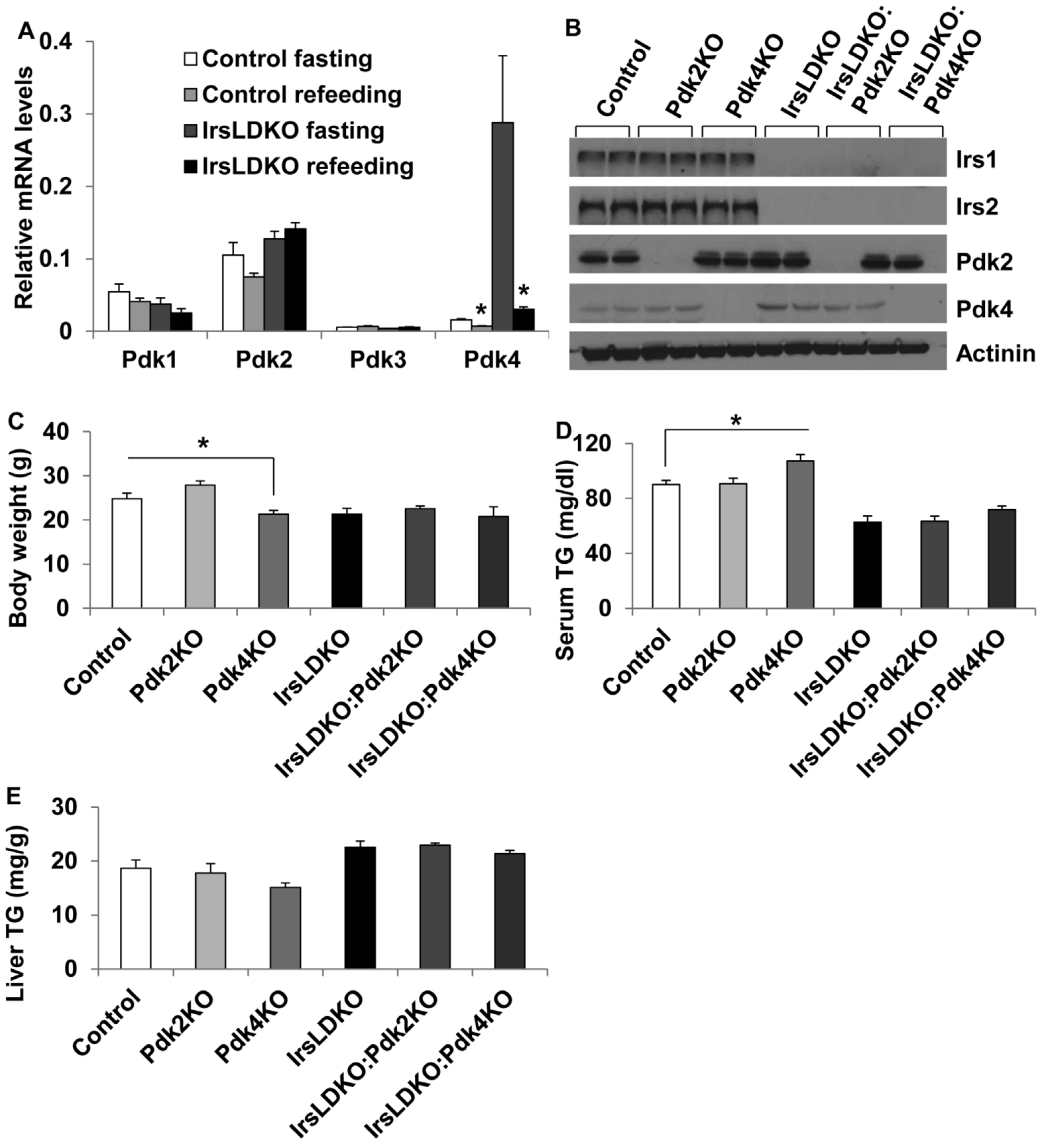
Knockout of the *Pdk* genes in wild-type and IrsLDKO mice. A, Control wild-type and IrsLDKO mice (n = 3) were fasted overnight for 16 hours and half of them were fed for 4 hours immediately after the fasting. *Pdks* gene expression in the liver was analyzed by real-time PCR and data were normalized to an internal control gene — Ppia. B, Western blot analysis of liver lysates from control and knockout mice. C, Body weight measurements in control and knockout mice (n = 6–20). D, Serum triglycerides (TG) were measured in overnight fasted control and knockout mice (n = 6–8). E, Liver TG analysis in control and knockout mice (n = 6–8). Pdk2KO, Pdk2 knockout; Pdk4KO, Pdk4 knockout; IrsLDKO, Irs1/2 liver-specific double knockout. Data are presented as means ± SEM. *, *P*<0.05 relative to corresponding controls.

**Figure 2:**
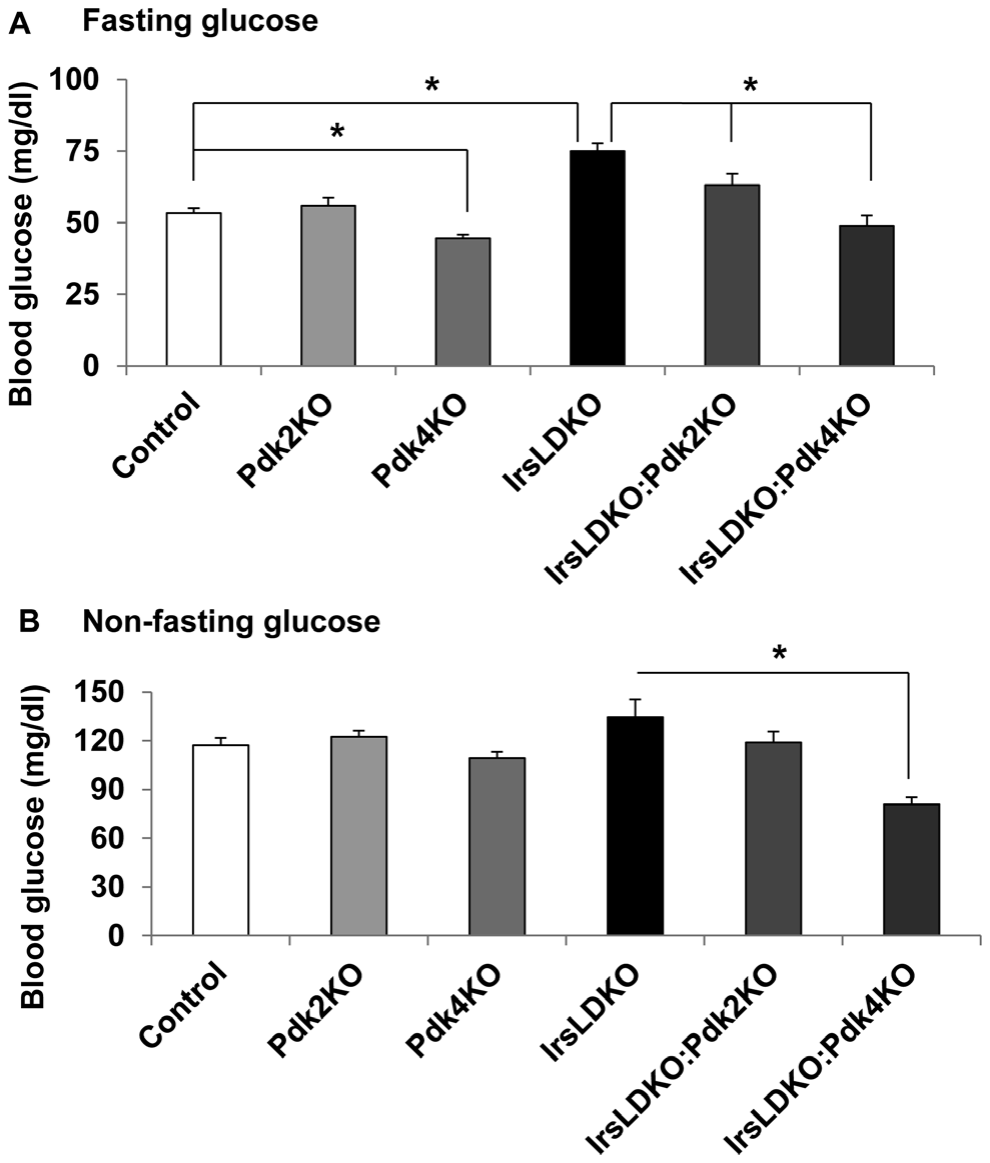
Deletion of the *Pdk4* gene improves hyperglycemia in IrsLDKO mice. A, Blood glucose was measured in overnight fasted control and knockout mice. B, Blood glucose was measured in *ad libitum* fed control and knockout mice. Data are presented as means ± SEM, n = 8–23. *, *P*<0.05 relative to corresponding controls.

**Figure 3:**
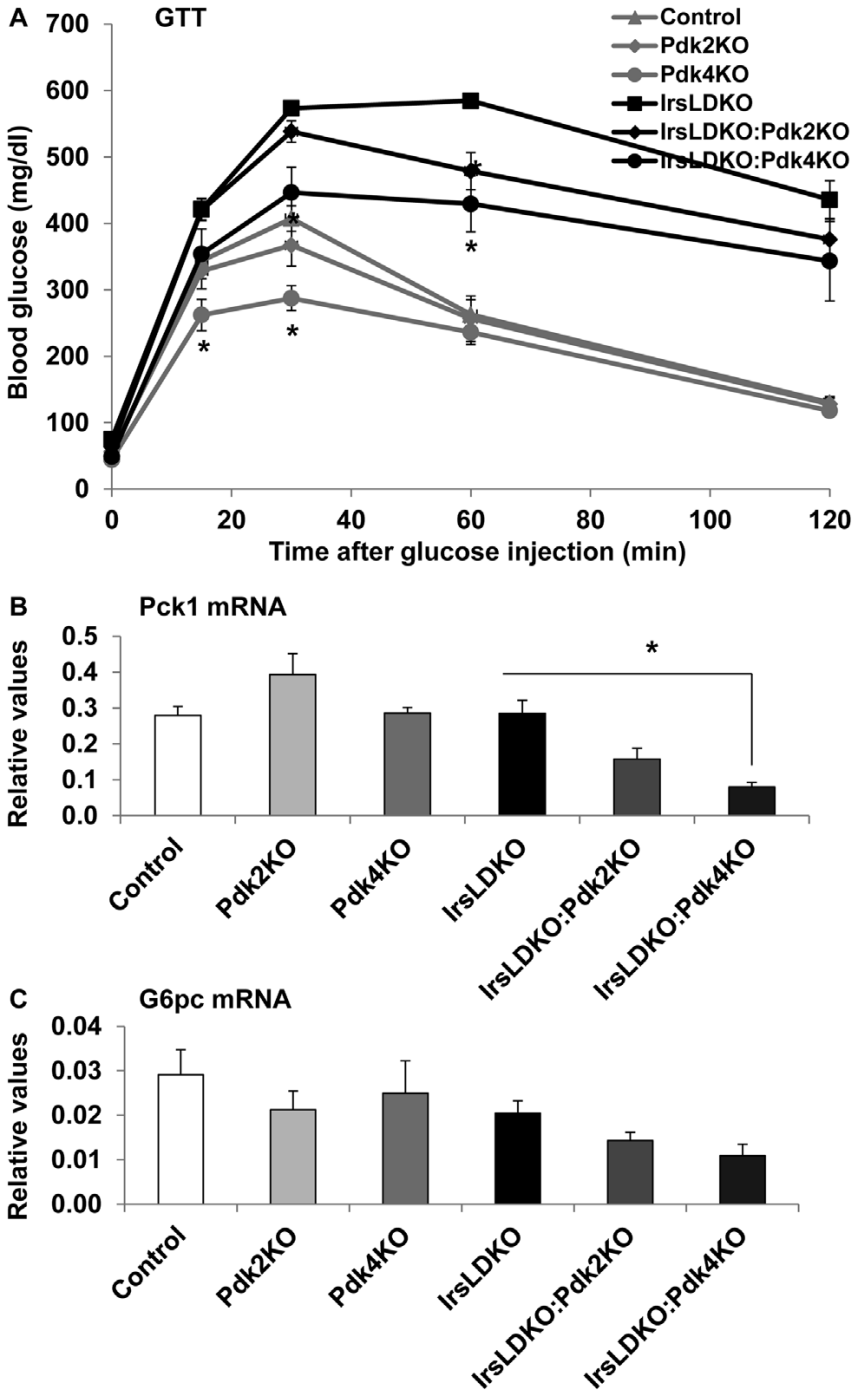
Ablation of Pdks improves glucose tolerance in IrsLDKO mice. A, Glucose tolerance tests (GTT) were performed in age-matched control and knockout mice (n = 8–12). B and C, Expression of gluconeogenic genes *Pck1* and *G6pc* was analyzed in the liver of overnight fasted control and knockout mice (n = 3). Data are presented as means ± SEM. *, *P*<0.05 relative to corresponding controls.

It has been previously reported that hepatic Irs1 and Irs2 play a crucial role in glucose homeostasis because simultaneous deletions of both genes in the liver (IrsLDKO) lead to diabetes in mice [20], [21]. Since Pdks can promote hepatic gluconeogenesis, it is possible that they contribute to the development of hyperglycemia in the IrsLDKO mice. To test this hypothesis, we first analyzed expression of all four *Pdk* genes in the liver of control wild-type and IrsLDKO mice after an overnight 16-hour fasting or immediately followed by 4-hour refeeding. According to mRNA analysis, Pdk2 is the most abundant among four Pdks in the liver of wild-type mice whereas Pdk4 was induced most in the fasted IrsLDKO livers (Figure 1A). Interestingly, refeeding could still suppress the hepatic *Pdk4* gene expression in the IrsLDKO mice (Figure 1A). To further investigate the role of Pdk2 and Pdk4 in the pathogenesis of diabetes in the IrsLDKO mice, we deleted either the *Pdk2* or *Pdk4* gene on the IrsLDKO genetic background (Figure 1B). While *Pdk4* knockout mice were significantly smaller than control wild-type mice, *Pdk4* deletion had no effect on the body weight of growth-retarded IrsLDKO mice (Figure 1C). Neither *Pdk2* nor *Pdk4* deletion had any significant effect on serum or hepatic triglycerides in the IrsLDKO mice (Figure 1, D and E). We then monitored blood glucose levels in control wild-type, single, double, and triple knockout mice. Deletion of *Pdk2* or *Pdk4* on the IrsLDKO genetic background (IrsLDKO:Pdk2KO and IrsLDKO:Pdk4KO, respectively) significantly lowered blood glucose levels under the fasting conditions (Figure 2A). In contrast, only IrsLDKO:Pdk4KO mice showed a significant decrease in blood glucose levels under the non-fasting conditions (Figure 2B). Additionally, deletion of either *Pdk2* or *Pdk4* also improved glucose metabolism during glucose tolerance tests, but ablation of the *Pdk4* gene had a more significant effect (Figure 3A). Gene expression analysis also revealed a significant decrease in *Pck1* (phosphoenoylpyruvate carboxykinase 1) but not *G6pc* (glucose-6-phosphatase, catalytic) mRNAs, suggesting that hepatic gluconeogenesis might be reduced in the IrsLDKO:Pdk4KO mice (Figure 3, B and C).

### Deletion of *Pdk2* or *Pdk4* improves insulin resistance in IrsLDKO mice

**Figure 4:**
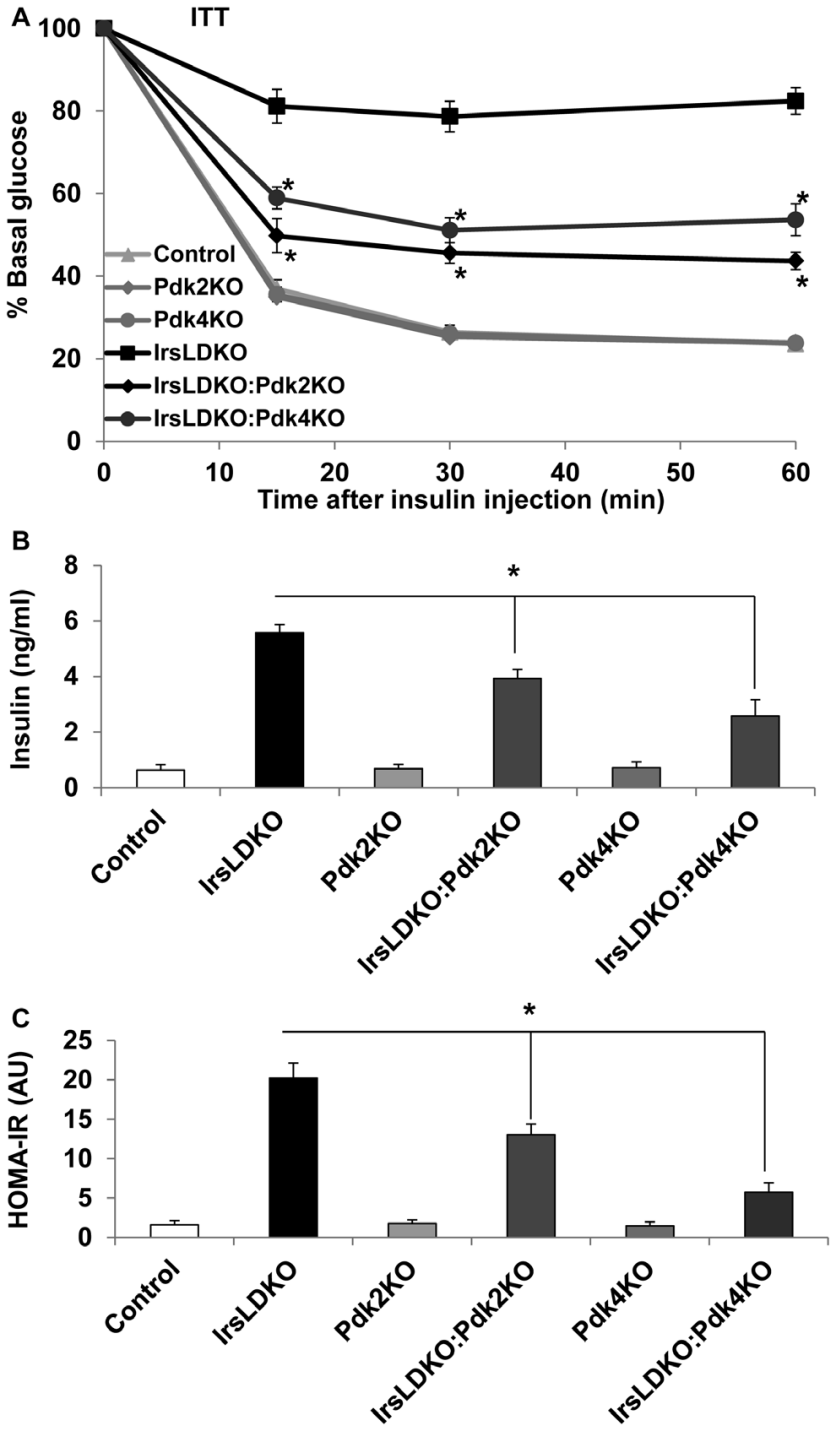
Inactivation of Pdks improves insulin sensitivity in IrsLDKO mice. A, Insulin tolerance tests (ITT) were performed in age-matched control and knockout mice (n = 8–20). B, Fasting plasma insulin was analyzed in age-matched control and knockout mice (n = 5–9). C, HOMA-IR (homeostatic model assessment-insulin resistance) was analyzed using fasting glucose and insulin data. Data are presented as means ± SEM. *, *P*<0.05 relative to corresponding controls.

**Figure 5:**
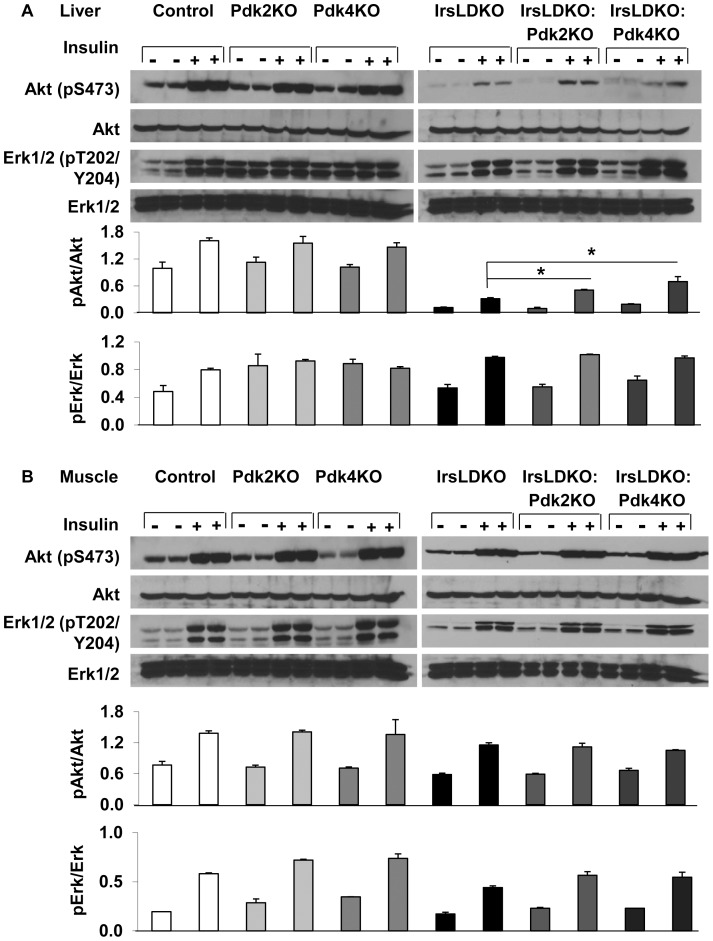
Insulin signaling analysis in the control and knockout mice. A and B, Animals were stimulated with 5 units of human insulin (saline as a vehicle control) for 3 min before liver and skeletal muscle samples were collected for Akt and Erk phosphorylation analyses. Western blot signals were quantified using the Quantity One software (Bio-Rad). Data are presented as means ± SEM. *, *P*<0.05 relative to corresponding controls.

To examine whether *Pdk2* or *Pdk4* knockout might affect insulin resistance in IrsLDKO mice, we performed insulin tolerance tests. The data showed that either *Pdk2* or *Pdk4* gene deletion remarkably improved insulin tolerance in the IrsLDKO mice (Figure 4A). In addition, fasting plasma insulin levels were also significantly reduced in IrsLDKO:Pdk2KO and IrsLDKO:Pdk4KO mice (Figure 4B). The HOMA-IR (homeostatic model analysis-insulin resistance) analysis indicated improved insulin resistance in those triple knockout mice (Figure 4C). In order to understand molecular changes during insulin action, we also analyzed insulin signaling in the liver and skeletal muscle. Akt phosphorylation (Ser473) was moderately elevated in the liver of IrsLDKO:Pdk2KO and IrsLDKO:Pdk4KO mice, and Erk1/2 phosphorylation (Thr202/Tyr204) was not significantly changed in the IrsLDKO:Pdk4KO or IrsLDKO:Pdk2 livers as compared to IrsLDKO livers (Figure 5A). No significant changes in insulin signaling were observed in the skeletal muscle of IrsLDKO:Pdk2KO or IrsLDKO:Pdk4KO mice in comparison to IrsLDKO mice (Figure 5B).

### Liver-specific knockdown of *Pdk2* or *Pdk4* in IrsLDKO mice

**Figure 6:**
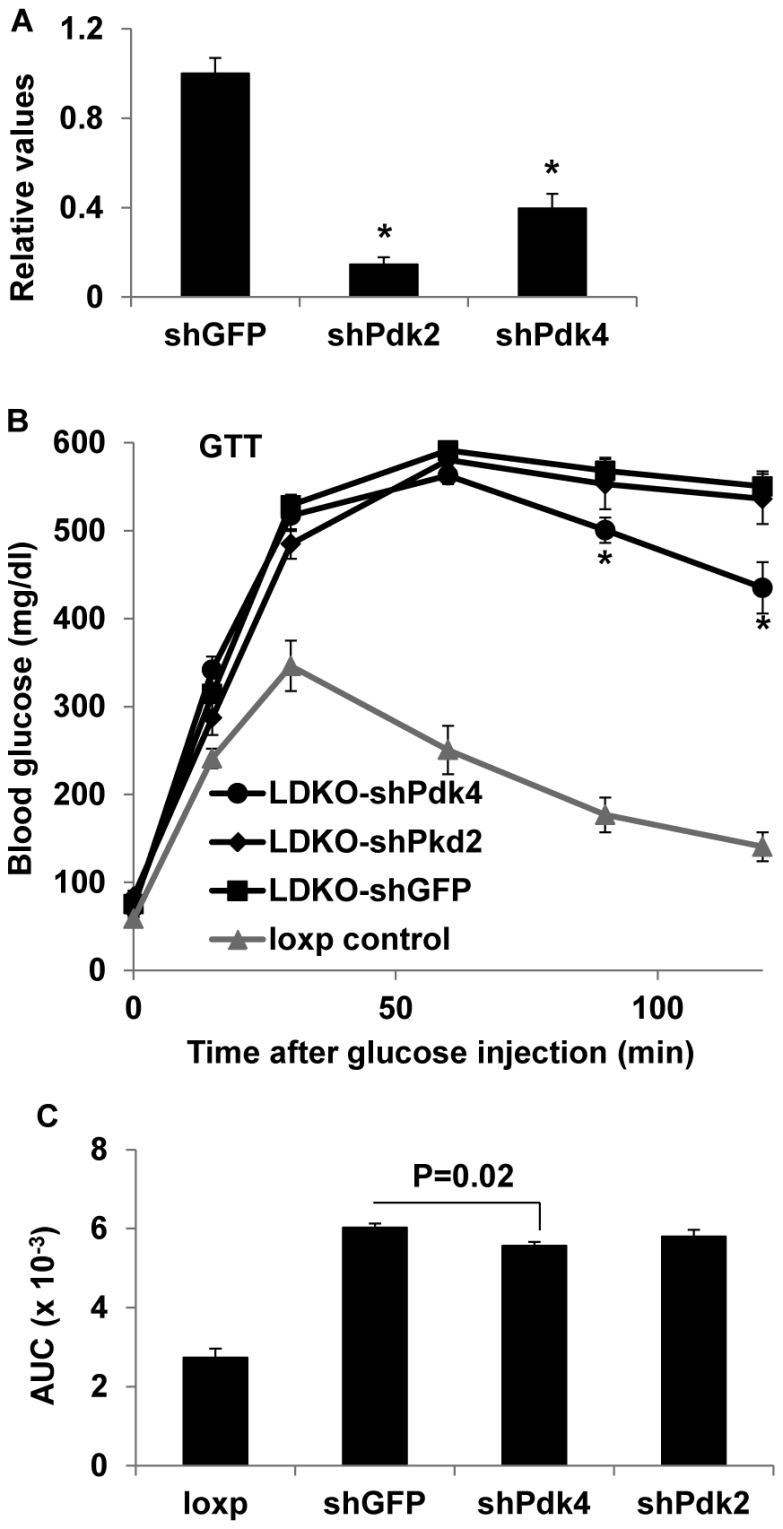
Hepatic Pdk4 knockdown moderately improves glucose tolerance in IrsLDKO mice. A, Gene knockdown efficiency was analyzed by real-time PCR in IrsLDKO livers transduced with shRNA adenoviruses against GFP (shGFP), Pdk2 (shPdk2), or Pdk4 (shPdk4). B, Glucose tolerance tests were performed in shRNA adenoviruses infected IrsLDKO mice. C, Area under curve analysis (AUC) was performed for the above glucose tolerance test data. Data are presented as means ± SEM, n = 4–5. *, *P*<0.05 relative to corresponding controls.

**Figure 7:**
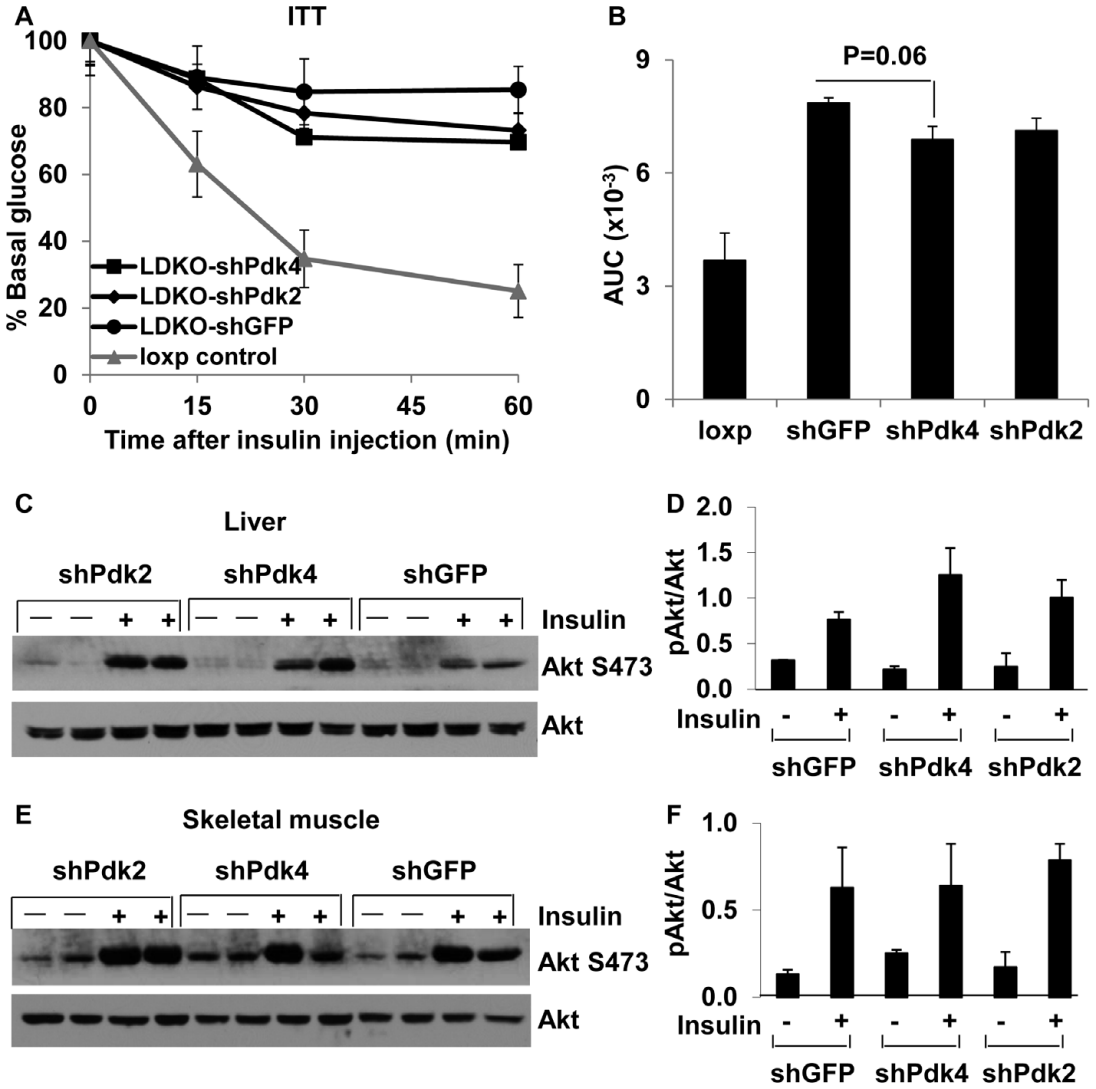
Pdk2 or Pdk4 knockdown has no significant effect on insulin tolerance in IrsLDKO mice. A, Insulin tolerance tests were performed on IrsLDKO mice (n = 4–5) that were injected with shGFP, shPdk2, and shPdk4 adenoviruses. B, Area under curve was analyzed for the above ITT data. C–F, Akt phosphorylation was analyzed in the liver and skeletal muscle of mice injected with shGFP, shPdk2, and shPdk4 adenoviruses. Western blot signals were also quantified using the Quantity One software. Data are presented as means ± SEM. *, *P*<0.05 relative to corresponding controls.

In order to directly assess the role of hepatic Pdks in glucose homeostasis, we used adenovirus-mediated shRNAs to knock down *Pdk2* or *Pdk4* specifically in the liver. Pdk2 and Pdk4 mRNA levels were reduced 85% and 60%, respectively (Figure 6A). Interestingly, although the Pdk4 knockdown efficiency was less than that of Pdk2, glucose tolerance was improved only in the Pdk4 knockdown mice in the last two time points during the glucose tolerance tests and the area under curve was significantly decreased in the Pdk4 knockdown group (Figure 6, B and C). Insulin tolerance tests did not reveal a significant improvement in either Pdk2 or Pdk4 knockdown mice although Pdk4 knockdown had a trend of improvement of insulin resistance (Figure 7, A and B). Insulin signaling analysis did not reveal any significant improvement in Akt phosphorylation in the liver or skeletal muscle of shPdk2 or shPdk4 mice as compared to shGFP control mice (Figure 7, C–F).

## Discussion

In this study, we directly assessed the involvement of Pdk2 and Pdk4 in the pathogenesis of diabetes. As Pdks regulate glucose homeostasis at least in two ways — inhibiting glycolysis and promoting gluconeogenesis, deletion of *Pdk* genes is expected to improve hyperglycemia and glucose tolerance. Indeed, both *Pdk2 and* Pdk4 ablations can improve glucose tolerance in the IrsLDKO mice, but only the *Pdk4* gene inactivation lowers both fasting and non-fasting glucose levels whereas the *Pdk2* gene deletion only decreases fasting blood glucose. This is quite intriguing because both *Pdk2* and *Pdk4* genes are ubiquitously expressed in most tissues and Pdk2 has been shown to be more potent for the inhibition of the PDC activity [3], [4], [17]. However, Pdk2 has also been shown to be more sensitive than other Pdks in response to allosteric regulators like pyruvate, NADH, and acetyl-CoA [24], [25]. Under non-diabetic conditions, Pdk2 deficiency causes a modest decrease in fed glucose levels whereas Pdk4 deficiency results in lower fasting glucose levels in mice as compared to the wild-type controls [17]. The differential effects can also be attributable to their respective modulation of the PDC activity since Pdk2 deficiency leads to increased PDC activity only in a fed state and Pdk4 deficiency affects the PDC activity in both fed and fasting states [17]. It seems that Pdk2 mainly regulates glucose utilization whereas Pdk4 may be involved in both hepatic gluconeogenesis and systemic glucose metabolism. In diabetic IrsLDKO mice that have severe hepatic insulin resistance, although glucose disposal may be decreased, the unsuppressed hepatic glucose production may be the major cause of hyperglycemia [26]. Under this condition, inactivation of the *Pdk4* gene produces a better effect than that by the *Pdk2* gene deletion largely because of a stronger role of Pdk4 in hepatic gluconeogenesis. This interpretation is supported by our data of the better glucose tolerance in the IrsLDKO:Pdk4 mice and the better insulin-stimulated glucose metabolism in the IrsLDKO:Pdk2 mice.

Although IrsLDKO mice are only deficient in hepatic Irs1 and Irs2, they manifest systemic insulin resistance as well [20], [21], which is indicated by decreased phosphorylation of Akt and Erk in the skeletal muscle (Figure 5B). In addition to the liver, the role of Pdks in other tissues including skeletal muscle and fat may be also critical for glucose homeostasis. This interpretation is consistent with our liver-specific knockdown of the *Pdk4* gene since the *Pdk4* knockdown only results in moderate changes in glucose tolerance in the IrsLDKO mice. But we should caution not to over-interpret the data due to the less ideal knockdown efficiency for the *Pdk4* gene.

The importance of Pdk4 in metabolism is evidenced by its dynamic gene expression in response to numerous factors, including insulin, glucocorticoid, fatty acids, bile acids, thyroid hormone, angiotensin II, retinoic acids, prolactin, growth hormone, adiponectin, epinephrine, thiazolidinediones, fibrates, statins, metformin, and others [7], [8], [13], [14], [22], [27]–[40]. Moreover, *Pdk4* gene expression is often induced in the liver and skeletal muscle under insulin resistance and diabetes conditions [6]–[15]. From this and other gene knockout studies [17], [18], [41], [42], it seems likely that a selective inhibition of the Pdk4 activity may be useful to normalize glucose metabolism and improve insulin resistance.

In summary, hepatic *Pdk4* gene expression is highly induced in diabetes. Inactivation of the *Pdk4* gene can improve hyperglycemia, glucose tolerance, and insulin resistance in diabetic mice. Overall, our data suggest that Pdk4 may be a useful therapeutic target for type 2 diabetes.
